# Prognostic value of left atrial strain in acute and chronic heart failure: A meta‐analysis

**DOI:** 10.1002/ehf2.15302

**Published:** 2025-04-20

**Authors:** Maria Concetta Pastore, Mariangela Vigna, Andrea Saglietto, Maria Alma Iuliano, Giulia Elena Mandoli, Andrea Stefanini, Chiara Carrucola, Laura Fusini, Luna Cavigli, Flavio D'ascenzi, Marta Focardi, Serafina Valente, Matteo Cameli

**Affiliations:** ^1^ Department of Medical Biotechnologies, Division of Cardiology University of Siena Siena Italy; ^2^ Cardiovascular and Thoracic Department, Division of Cardiology Hospital Citta Della Salute e della Scienza di Torino Turin Italy; ^3^ Centro Cardiologico Monzino IRCCS Milan Italy

**Keywords:** Acute, Chronic, Heart failure, Left atrial strain, Prognosis, Speckle tracking

## Abstract

**Aims:**

Heart failure (HF) is a global health burden which prognostic assessment is currently challenging. Speckle tracking left atrial strain is widely recognized as a predictor of HF outcome. Our aim was to systematically investigate the prognostic value of peak atrial longitudinal strain (PALS) in acute and chronic HF and according to left ventricular (LV) function, age and gender.

**Methods and results:**

A systematic literature search of medical databases was performed using PRISMA principles. All relevant studies reporting the prognostic value of LA strain in HF with reduced, mildly reduced and preserved ejection fraction (EF) with ≥6 months follow‐up were included. All‐cause mortality and HF hospitalization were considered as primary endpoint. Random‐effect meta‐analysis was performed to evaluate the pooled hazard ratios (HR) of the primary outcome. Eight studies (*n* = 5767 patients, median [interquartile range] age = 66.3 [65; 68.6]) satisfied the inclusion criteria (five chronic HF, two acute HF and one both). Median global PALS was 17.6 [14.9; 26.8]%, median LVEF was 36 [30; 56]%, median left ventricular global longitudinal strain (GLS) was −9% [−7; −16.9]. Over a median follow‐up of 903 [321; 1062] days, 2688 patients reached the primary endpoint (944 all‐cause mortality and 1963 hospitalizations). Each unit decrease in global PALS was independently associated with 5% increase for the primary endpoint (meta‐analytic HR = 1.05; 95% CI [1.02–1.07]; *P* < 0.01). Subgroup analysis showed no differences in acute and chronic HF (*P* = 0.18). Meta‐regression analysis showed a higher prognostic value of global PALS for lower values of LVEF (beta = −0.0023).

**Conclusions:**

Global PALS may be used as prognostic tool in acute and chronic HF and especially in patients with reduced EF, providing an additional independent value for risk stratification in clinical practice.

## Graphical abstract

The prognostic evaluation of heart failure (HF) beyond ejection fraction remains challenging. Moreover, reliable prognosticators in chronic HF sometimes lose their value in acute settings or vice versa. Speckle tracking echocardiography offers new sensitive parameters for prognostic stratification in heart failure with reduced and preserved ejection fraction. This meta‐analysis shows that left atrial strain is an independent and robust prognostic predictor both in acute and chronic heart failure, particularly for lower values of ejection fraction.

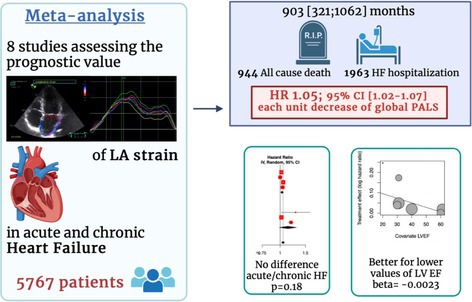

## Introduction

Heart failure (HF) is a clinical syndrome consisting of typical symptoms that may be accompanied by signs, characterized by variable aetiologies, high morbidity and mortality and often challenging diagnosis and treatment.^1^

While the diagnosis of chronic heart failure (CHF) requires the presence of symptoms and/or signs of heart failure (HF) and objective evidence of cardiac dysfunction, acute heart failure (AHF) refers to rapid onset of symptoms and/or signs of HF and may either be the first manifestation of HF (new onset) or, more frequently, represent an acute decompensation of chronic HF, with rapid progression and potential life‐threatening consequences. Therefore, in these two pathological entities an accurate prognostic stratification is pivotal to guide treatment. In patients with acute HF an early intervention becomes crucial to restore haemodynamic compensation and stabilize patients' symptoms. On the other hand, in patients with chronic HF, the optimization of therapeutic resources is fundamental to avoid haemodynamic decompensation and hospitalizations as multiple readmissions are invariably associated with poor prognosis.^2^

Even if HF has traditionally been considered as a disease pertaining to the left ventricle (LV), in the last decades, more attention has been given to the involvement of left atrium (LA) in HF.

The LA contributes to cardiac haemodynamics by modulating LV left ventricular (LV) filling through the interplay of atrial reservoir, conduit and booster function.^3^, ^4^

These phases of LA function may be easily quantified by speckle tracking echocardiography (STE) that provides LA deformation analysis all over the cardiac cycle; peak atrial longitudinal strain (PALS) is the measure of LA reservoir function and is the most used in clinical practice. PALS has been proposed as an additional index to include in the currently recommended algorithm for the detection of diastolic dysfunction to better classify those patients in the ‘grey zone’ of undetermined ‘diastolic function’.^5^

In fact, global PALS has shown to be an accurate index of LV filling pressures, to have a strong association with elevated pulmonary capillary wedge pressures both in patients with HF with reduced ejection fraction (HFrEF) and heart failure with preserved ejection fraction (HFpEF)^6^ and to be a superior prognosticator in HF beyond ejection fraction over conventional echocardiographic parameters.^7^

The aim of our meta‐analysis was to systematically investigate the prognostic value of PALS for cardiovascular (CV) events in HF in acute and chronic HF and according to LV function, age and gender.

## Methods

### Data sources and search strategy

The following systematic review was conducted in strict adherence to the Preferred Reporting Items for Systematic Reviews and Meta‐analysis (PRISMA) guidelines. Comprehensive searches were conducted across various databases (including Pubmed, Scopus, Ovid Online, EMBASE, Web of Science and Cochrane Central Register) to retrieve pertinent information from inception to October 2023. The following keywords and Boolean operators were used as search terms in all databases, following different searching steps:

1. ‘heart failure’ OR ‘heart insufficiency’ OR ‘failing heart’;
2. ‘left atrial,’ OR ‘left atrium’ AND speckle tracking as: ‘speckle’ AND ‘tracking’ OR ‘strain’ OR ‘deformation’
3. ‘prognosis‘ OR ‘outcome’ OR ‘mortality’ OR ‘survival’ OR ‘survival’
4. ‘Echocardiography’ OR ‘echocardiogram’.

References of included articles were manually searched to identify additional eligible studies.

All studies in English language reporting the predictive value of atrial strain (LA strain) for mortality and/or cardiovascular events (cardiovascular death, hospitalization for heart failure, heart transplantation and ventricular assist device implantation) in patients with reduced ejection fraction (HFrEF), moderately reduced ejection fraction (HFmrEF) and preserved ejection fraction (HFpEF) were included, with a follow‐up period of at least 6 months.

### Inclusion and exclusion criteria

The meta‐analysis included studies involving patients with acute and chronic heart failure according to the latest European society of cardiology (ESC) guidelines,^1^ both those with reduced ejection fraction (HFrEF) or mildly reduced ejection fraction (HFmrEF) with documented EF < 50% and those with preserved ejection fraction (HFpEF) with documented EF > 5LV0%. These studies provided quantitative data (mean with standard deviation or standard error) on speckle tracking parameters, mandatory was PALS, then GLS, fwRVLS, RV GLS and right atrial strain as optional, obtained by echocardiography. The mandatory information was prognostic outcome, encompassing all‐cause mortality, cardiovascular mortality and heart failure‐related hospitalizations. These studies were required to have a follow‐up period for the primary endpoint of at least 6 months, with additional information available for at least 1‐year follow‐up.

All studies that did not meet these criteria were excluded as they were not relevant to the objective of the review. Additionally, case reports, case series and conference abstracts were excluded as they were not available in full form.

### Endpoint

The aim of this meta‐analysis is to conduct a systematic investigation into the prognostic value of peak atrial longitudinal strain (PALS) for all‐cause mortality or HF re‐hospitalization in HF. Additionally, it examines variations in this value in the context of acute/chronic HF, as well as in relation to left ventricular (LV) function and patient age.

The primary endpoint is represented by all‐cause mortality and hospitalizations for heart failure.

### Data extraction

Three independent authors have independently extracted data related to the publication of the study (author names, journal, publication year, number of pages, DOI and publication status), the study type (prospective or retrospective, case–control, cohort study, cross‐sectional, monocentric, bicentric or multicentric), follow‐up duration, classification of heart failure (chronic or acute, HFpEF, HFmEF and HFrEF), population characteristics (outpatient and inpatient), study exclusion criteria, primary and secondary endpoints, the type of echocardiography machine used (GE, Philips, etc.), the software used for STE analysis (EchoPAC, Tomtec, etc.), the reference type for atrial strain assessment (Q wave or P wave), PALS values assessed in four chambers and two chambers, statistical analysis (univariate or multivariate, ROC curves, Kaplan–Meier curves and correlation analysis), the number of study patients, the number of patients experiencing all‐cause mortality, cardiovascular mortality, major cardiovascular events, hospitalization for heart failure at a 1‐year follow‐up, basic and advanced echocardiographic parameters, population characteristics and clinical variables.

### Echocardiography

All patients included in the studies underwent a standard echocardiographic evaluation performed by experienced physicians or sonographers using commercially available ultrasound systems. In the two studies including acute HF patients, echocardiography was performed within 48 h after admission.^2^, ^10^

In most studies, speckle tracking has been recorded through standard 2D grayscale acquisitions with a frame rate exceeding 50/s. Left atrial strain was calculated using various types of software. Common steps include delineating a region of interest (ROI) and automatically tracking speckle points from frame to frame in the apical four‐chamber and two‐chamber views.

Peak positive longitudinal strain at the end of the left atrial filling phase is defined as PALS. The reference point used is the QRS complex.^3^ Setting the QRS complex as the reference, atrial strain is represented with two peaks, corresponding to different phases of atrial function. The parameters measured through speckle tracking analysis of the left atrium are PALS, measured at the end of the atrial reservoir phase, and peak atrial contraction strain (PACS), identified just before the onset of the active atrial contraction phase.

### Statistical analysis

Baseline characteristics of pooled study populations were reported as median values between the included studies, along with their interquartile range (IQR). Meta‐analysis of the hazard ratio (HR) per unit increase [along with its 95% confidence interval (CI)] of the overall PALS variable was calculated using a random effects model in order to investigate the association between the overall PALS variable and the considered outcome of all‐cause mortality or HF hospitalization. *Table* *1* lists all the variables included in the multivariate analysis in each study. For studies with different primary outcome, the HR related to the outcome of mortality was used for the analysis. Forest plot for the outcome were also reported. The *I* ^2^ measure was used to assess statistical heterogeneity among studies. *I* ^2^ values of 25%, 50% and 75% were represented as low, moderate and high heterogeneity, respectively. To anticipate heterogeneity due to different types of cohorts of all studies, we chose to apply random effects model for the analysis, which accounts for heterogeneity. We conducted a subgroup analysis to determine the impact of PALS on prognosis in patients with acute and chronic HF. Then, meta‐regression analysis was applied to investigate the potential influence of LV ejection fraction, age and LV global longitudinal strain on the association of PALS with outcome. To assess publication bias, funnel plot and Egger's test were chosen to examine the study distribution. The trim and fill analysis were further used to evaluate theoretical missing research studies because of publication bias. RStudio version 1.3 was used to perform statistical analysis. A *P*‐value of <0.05 was considered statistically significant.

**Table 1:** Variables included in the multivariate analysis considered for the calculation of the hazard ratios in each study evaluated in this meta‐analysis

Study	Variables included in the adjusted Cox proportional hazard models
Freed BH et al.	Sex Atrial fibrillation MAGGIC risk score (includes age, LV ejection fraction, creatinine, diabetes, chronic obstructive pulmonary disease, systolic blood pressure, body mass index, heart rate, NYHA functional class, ACE‐inhibitor use, beta‐blocker use, heart failure duration and current smoking) LV mass LA volume Average E/E′ ratio LV longitudinal strain RV free wall strain PALS
Santos AB et al.	Age Sex Race Randomization strata Enrolment region (Americas vs. Russia/Georgia) Randomized treatment assignment History of atrial fibrillation Heart rate New York Heart Association class History of stroke Creatinine Haematocrit Left ventricular ejection fraction Left atrial volume index Left ventricular global longitudinal strain PALS
Malagoli et al.	Age NYHA class GFR BNP LVESVi LVEF LAVimax E/A ratio Average E/e′ ratio PALS
Carluccio et al.	EMPHASIS‐HF risk score (including male sex, diabetes, prior hospitalization for HF, prior MI/CABG, age, SBP, BMI, HR, eGFR and haemoglobin) NYHA class Log (BNP) ICD at baseline CRT implant during follow‐up LAVI LVEDVI LVEF E/E′ ratio Mitral regurgitation severity Left ventricular global longitudinal strain
Overall‐Park et al.	Age Sex BMI NYHA dBP HR Hypertension Diabetes mellitus Atrial fibrillation Ischaemic heart disease Haemoglobin Creatinine Total cholesterol LVEF logNTproBNP LAVI PALS
Pastore et al.	Age NT‐proBNP LAVI LV EF TAPSE LV GLS E/E′
Deferm et al.	LAVI LV end‐diastolic volume LV GLS

ACE, angiotensin‐converting‐enzyme; BMI, body mass index; BNP, brain natriuretic peptide; CABG, coronary artery bypass grafting; CRT, cardiac resynchronization therapy; dBP, diastolic blood pressure; E/A, E wave/A wave by pulsed wave Doppler; E/E’, E wave by pulsed‐wave Doppler/E’ wave by Tissue Doppler imaging ratio; HF, heart failure; HR, heart rate; GFR, glomerular filtration rate; GLS, global longitudinal strain; ICD, intracardiac cardioverter defibrillator; LA, left atrium; LAVI, left atrial volume index; LV, left ventricle; LVEF, left ventricular ejection fraction; LVEDVi, left ventricular end‐diastolic volume index LVESVi, left ventricular end‐systolic volume index; MI, myocardial infarction; NYHA, New York Heart Association; NTproBNP, N‐terminal‐pro‐brain natriuretic peptide; PALS, peak atrial longitudinal strain; RV, right ventricle; TAPSE, tricuspid annular plane systolic excursion.

## Results

### Study selection

**Figure 1:**
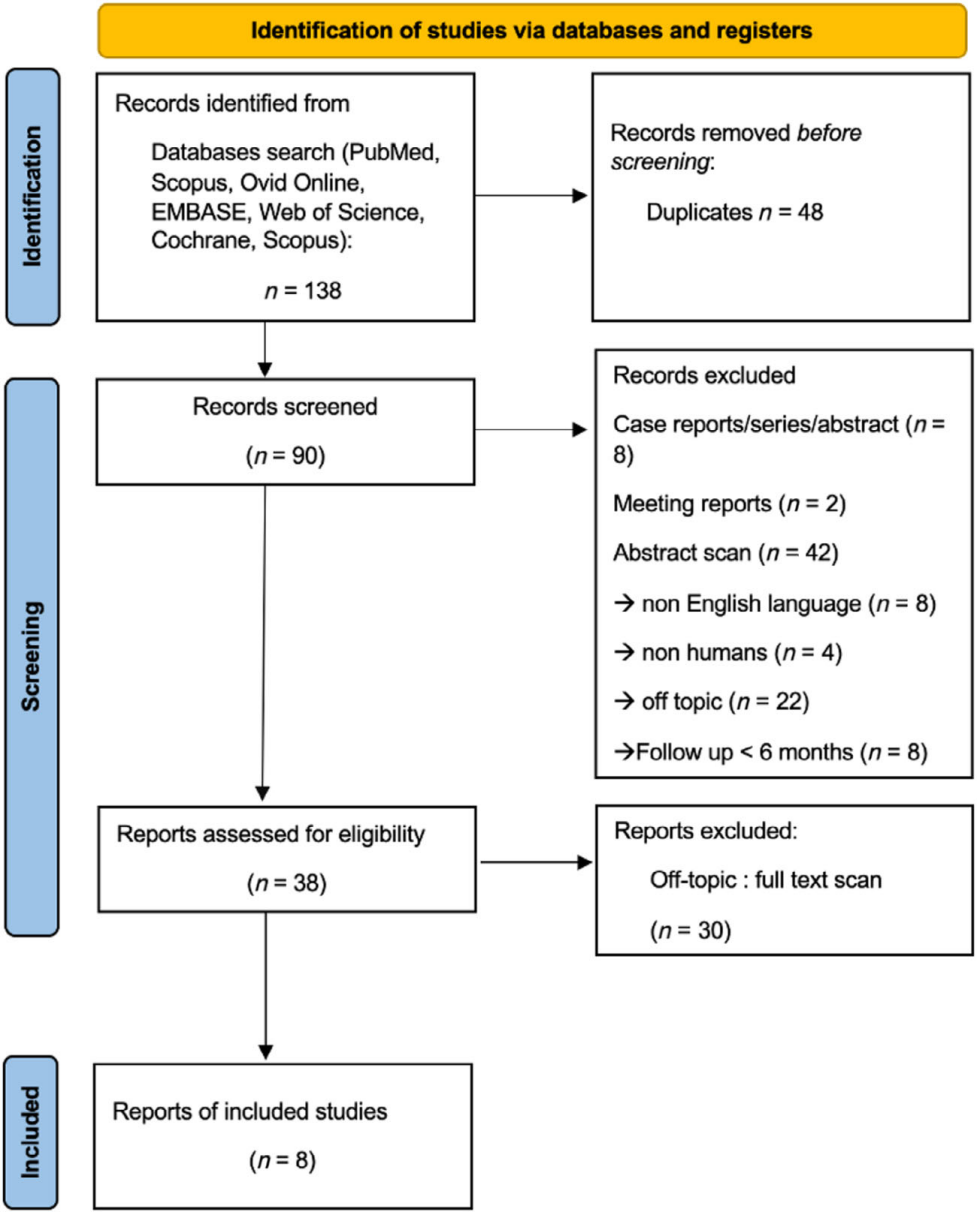
Study selection according to PRISMA guidelines.

From the initial pool of 138 articles selected following the search strategy, 48 articles were removed due to duplication. Additionally, 52 studies were excluded for not meeting the inclusion criteria. Among these, 8 were case reports or case series, or abstracts, 2 were conference reports, and 42 involved animals, were in a language other than English, or were off‐topic, or had a follow‐up period of less than 6 months. Consequently, a total of 38 potentially eligible studies were assessed after reviewing titles and abstracts. Following a comprehensive text review, 30 studies were excluded as they were deemed off‐topic. Therefore, a total of 8 studies, which included patients with heart failure, were analysed, of which 7 were included in the multivariate analysis, since one of the studies did not report the HRs for the prognostic evaluation.^6^ The selection process of the studies included is depicted in *Figure* *1*, while study design and patient characteristics are resumed in *Table* *2*.

**Table 2:** Study design and patient characteristics of all studies included in the present meta‐analysis

Study	Design	Study period	Follow up (days, median [IQR])	Type of HF	Ischaemic aetiology (%)	Chronic/acute HF	Age (years, mean ± SD)	Female (%)	Atrial fibrillation (%)	Hypertension (%)	Diabetes (%)	ACE‐i (%)	Beta‐blockers (%)	MRA (%)	Diuretics (%)	LV EF (mean ± SD)	Primary endpoint	Vendor used for STE analysis	E/E′ (mean ± SD)	LAVI (mL/m^2^, mean ± SD)	Global PALS (%, mean ± SD or median [IQR])	Mitral regurgitation >moderate (%)	LV GLS (%, mean ± SD or median [IQR])
Freed BH et al.	Prospective	March 2008 – May 2011	414 [135–717]	HFpEF	50	Chronic HF	65 ± 13	64	26	75	30	54	67	12	76.29	61 ± 6.4	CV hospitalization and death: hospitalization for any CV cause and death from any cause.	Tomtec	15 ± 8.1	34.4 ± 13.7	36.2 ± 14.9	14	−17.5 ± 4.1
Santos AB et al.	Prospective	August 2006 – January 2012	930 [540–1,290]	HFpEF	44.9	Chronic HF	68.9 ± 9.7	56.6	21.6	93	41.6	NA	NA	NA	NA	60.4 ± 7.7	Composite of CV death, HF hospitalization, and aborted sudden death	Tomtec	15.7 ± 6.7	28.4 ± 11.4	25.9 ± 7.7	11.7	−16.4 ± 3.4
Malagoli et al.	Prospective	September 2011 – March 2015	44 [31–58]	HFrEF	64	Chronic HF	67.37 ± 10.31	18.53	‐	68.88	24.47	86.01	93	37.06	74.82	31.83 ± 6.037	MACE: HF hospitalization, nonfatal MI, nonfatal stroke, and CV death	EchoPac	20.92 ± 11.46	46.78 ± 15.93	19.74 ± 9.25	Severe: 2.5	NA
Carluccio et al.	Prospective	January 2013 – December 2016	888 [393–1,539]	HFrEF	38	Chronic HF	65.2 ± 12.3	24	‐		26	88	79	NA	90	30 ± 5	All‐cause death/HF hospitalization	EchoPac	14 ± 4	52.6 ± 18.6	15.5 [11.2‐20.6]	Severe: 15	−8.3 ± 2.9
Overall‐Park et al.	Prospective	January 2009 – December 2016	918 [348–1,632]	HFrEF; HFpEF, HFmrEF	32.5	Acute HF	70.6 ± 13.8	47.2	29.6	57.5	34.4	NA	63	46	NA	40 ± 15.5	All‐cause mortality and HF hospitalization All‐cause death and HF hospitalization	Tomtec	18.9 ± 11	60.1 ± 39.5	14.7 ± 10.1	‐	NA
Overall‐Bouwmeester et al.	Prospective	December 2017 – September 2019	365	HFrEF; HFpEF, HFmrEF	39	Chronic	68 ±	31	‐	51	15	NA	39	31	41	44 ±	All‐cause death and HF hospitalization	Philips iE33 or Philips EPIQ, Andover, MA, USA	NA	NA	27 [20–35]	18	−8.3 ± 3.9
Pastore et al.	Retrospective	January 2015 – September 2020	1,460 [365–1825]	HFrEF; HFpEF, HFmrEF	46	Chronic HF and Acute HF	65 ± 12	37	‐	50	27	35	45	37	46	31 ± 9	All‐cause death and HF hospitalization	EchoPac, GE, Milwaukee, Wisconsin	14.3 ± 8	43.9 ± 21	15.5 [10.22]	41	−7.3 ± 3.5
Deferm et al.	Prospective	2011–2013	655	HFrEF	48.4	Acute HF	64 ± 15	23	32.3	48,4	29	NA	74	42	58	20 ± 12	All‐cause death and HF hospitalization	Image arena v4.6	16.8 ± 6.6	64 ± 25	8.8 ± 3	51.6	NA

CV, cardiovascular; EF, ejection fraction; HF, heart failure; HFmrEF, heart failure with mildly reduced ejection fraction; HFpEF, heart failure with preserved ejection fraction; HFrEF, heart failure with reduced ejection fraction; IQR, interquartile range; LAVI, left atrial volume index; LV, left ventricle; MACE, major adverse cardiac events; MI, myocardial infarction NA, not available; PALS, peak atrial longitudinal strain; SD, standard deviation; STE, speckle tracking echocardiography.

Eight studies (5767 patients, median [IQR] age = 66.3 [65; 68.6], 37% female) were included based on the selection criteria. Five studies were conducted in patients with chronic heart failure, two studies in patients with acute heart failure, and one study in both clinical settings. Among these, six studies included patients with HFrEF, three studies included patients with HFmrEF, and five studies included patients with HFpEF.

In all studies QRS was used as reference point for the calculation of LA strain and global PALS was calculated using both 4‐ and 2‐chamber view. The median value of global peak atrial longitudinal strain (PALS) was 17.6% [14.9; 26.8]. Median left ventricular ejection fraction (LVEF) was 36% [30; 56]. Finally, the median global longitudinal strain (GLS) of the left ventricle was −9% [−7; −16.9].

The median follow‐up period was 903 days [321; 1062]. Among the participants, 2688 patients reached the primary endpoint, with a total of 944 deaths from any cause and 1963 hospitalizations for HF.

With multivariate analysis, we found that every unit decrease in global PALS was independently associated with a 5% increase in the primary endpoint (meta‐analytic HR 1.05; 95% CI [1.02–1.07]; *P* < 0.01).

**Figure 2:**
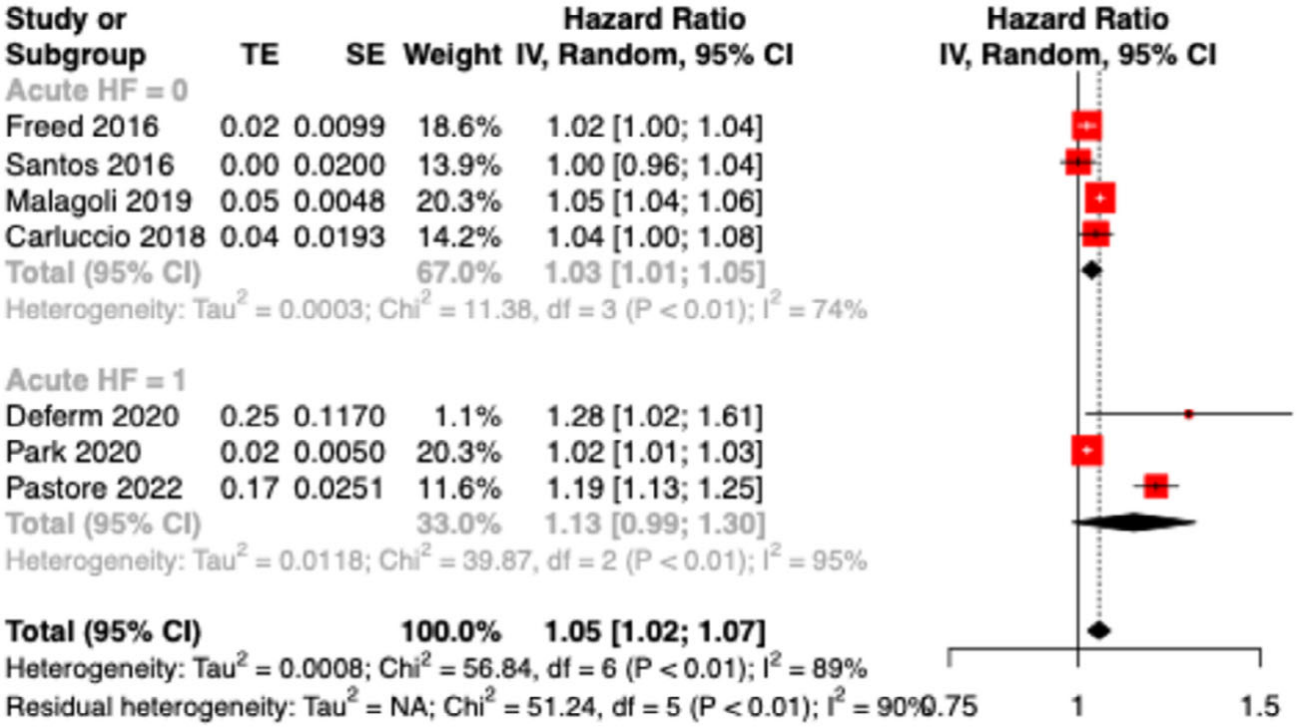
Subgroup analysis for acute (acute HF = 1) and chronic (acute HF = 0) heart failure. Hazard ratios (HR) are reported per unit decrease of global peak atrial longitudinal strain (PALS).

Subgroup analysis performed on patients with acute and chronic heart failure did not show significant differences (*P* = 0.18, *Figure* *2*).

**Figure 3:**
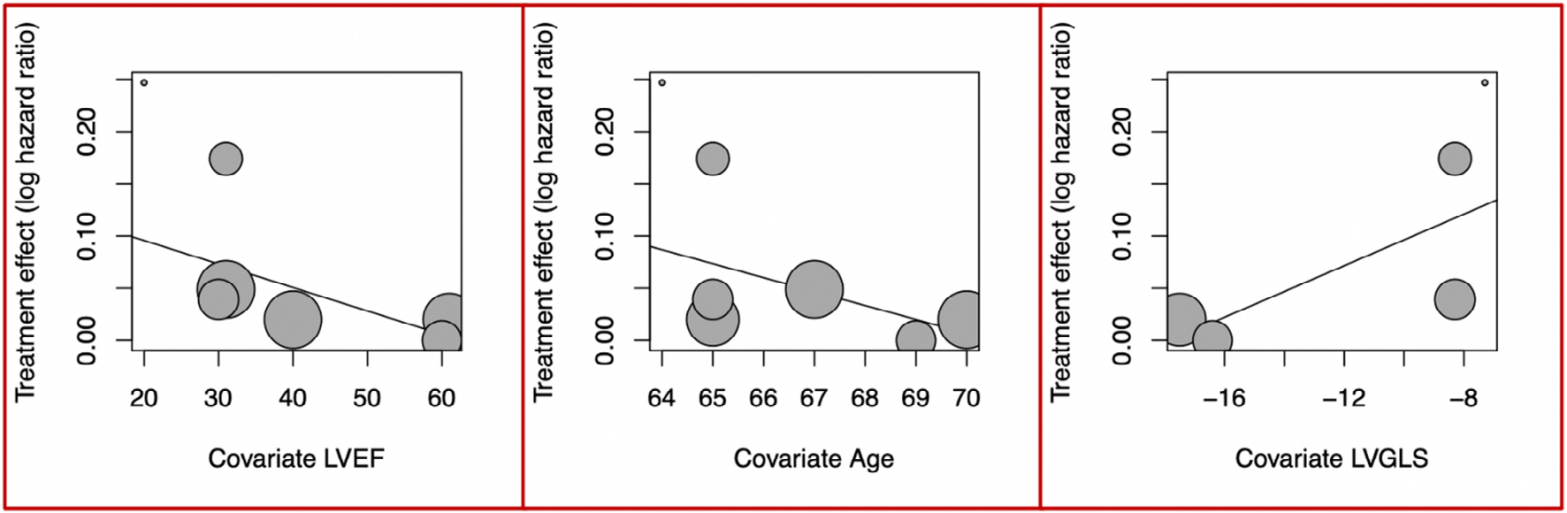
Meta‐regression analysis for left ventricular ejection fraction, age and left ventricular global longitudinal strain.

Meta‐regression analysis revealed that the prognostic value of global PALS was higher for lower values of left ventricular ejection fraction (LVEF) (beta = −0.0023). A similar trend was observed for worse global longitudinal strain (LV GLS) and younger age, although this trend did not reach statistical significance (*Figure* *3*).

### Publication bias

The funnel plot analysis did not reveal any evidence of publication bias (Egger's *P* = 0.45).

## Discussion

The present meta‐analysis was the first to demonstrate that global PALS maintains its strong prognostic value for HF patients both in acute and chronic settings. This is the first crucial point of our study, since many authors are often concerned on the load‐dependency limitation of strain parameters. In fact, patients with acute HF are characterized by the rapid development of haemodynamic overload, which may influence the quantification of LA deformation properties. However, in the last years, LA strain has emerged as an index of LV filling pressures, also validated using invasive measures and over basic echocardiographic indices (such as LAVi, E/E′ and PAPs). It is known that patients with higher LV filling pressures are prone to develop poor short‐term and long‐term outcome,^7^ therefore, it may be said that its usefulness in acute setting is emphasized by its load dependency, and it also suggests a potential value of this parameters for haemodynamic monitoring in in‐hospital decongestion protocols.

In acute HF, the reservoir and booster pump function are markedly impaired. Deferm et al. demonstrated that, after an adequate decongestive therapy and afterload reduction, LA reservoir function improved immediately, while LA contractile function improved incrementally up to 6 weeks after appropriate decongestion, even in advanced HF states.

Moreover, a significant association between PALS and NT‐proBNP in acute and chronic HF has been proved in a recent study by our group, in which we showed that PALS was a significant predictor of NT‐proBNP raise and an independent prognostic index for all‐cause mortality and HF hospitalization in patients with acute and chronic HF.^8^ This suggested its utility to assess prognosis early after HF hospitalization, and then serially to monitor haemodynamic re‐compensation in response to therapy. Also, Bouwmeester et al. showed that HF outpatients with LA strain ≤17% had significantly worse survival and higher re‐hospitalization rate and that LA strain was also inversely correlated with NT‐proBNP.^6^

Furthermore, Park et al. highlighted the prognostic role of PALS in patients with acute HF, regardless of HF phenotypes, age, sex, BMI, medications and blood markers.^9^

As concerning chronic HF, Carluccio et al. also observed the prognostic value of PALS ≤12.9% in a cohort of 405 HFrEF outpatients for HF hospitalization and all‐cause death.^10^

Also, among stable outpatients with chronic HFrEF studied by Malagoli et al., those with lower global PALS showed worse event‐free survival and developed AF more frequently than those with higher PALS.^11^

Our meta‐regression analysis found that the prognostic utility of LA strain is particularly useful in older patients with HFrEF and lower LV GLS, confirming previous evidence^11^, ^12^ in which, indeed, this parameter emerged more as a diagnostic marker for HFpEF and a prognostic index in HFrEF. This may be explained by two different pathophysiologic mechanism of HFrEF and HFpEF: in HFrEF, a primary damage of the LV is reflected on the other cardiac chambers, first on the LA and then on pulmonary circulation and RV, due to the raise in LV filling pressures parallel to HF progression^13^; whereas HFpEF derives from diastolic dysfunction, which is mainly characterized by preserved LV contractile performance and impaired LV relaxation, causing an increase in LV filling pressures which then affects LA performance before LV performance.^14^

A recent analysis conducted on a considerable number of HF patients at different stages of severity showed that, even though LV GLS is the strongest determinant of PALS, it was less likely to find patients with preserved PALS and reduced GLS than vice‐versa, and that PALS is the superior echocardiographic parameter for characterization of the HF stages and for predicting the presence of symptoms.^15^

Therefore, being the primary target of cardiac damage in HFpEF, LA strain has been suggested as a sensitive parameter for diagnosis and to recognize structural cardiac damage and diastolic dysfunction over conventional echocardiographic parameters in these patients^3^; in fact, it has been included in the latest EACVI recommendations among the parameters to assess for the diagnosis of HFpEF.^16^ On the other hand, many studies showed that LA strain is more useful as an index of the severity of the disease in HF patients, when patients also develop symptoms, as the reduction in LA deformation properties reflect the chronic (or the huge, in acute settings) increase in LV filling pressures deriving from a primary LV damage, and the extent of LA fibrosis.^17^

In two studies of our meta‐analysis, conducted in patients with HFpEF, the clinic and prognostic role of LA strain has been proved. Freed et al. showed the power of PALS to predict the risk of HF hospitalization and death beyond LV or LA structure, LV strain and RV strain.

By contrast, Santos et al. showed that global PALS was associated with older age, higher prevalence of AF and LV hypertrophy, worse LV and right ventricular and LV diastolic function. Although PALS was highly associated with a composite endpoint derived by cardiovascular death, HF hospitalization and aborted sudden death and with HF hospitalization alone, its prognostic value was not independent of LV strain and E/E' ratio, likely because these were determinant of the reduction of global PALS in this patient population.^18^ As hypothesized before, PALS is reduced in this group of patients, however, it is the result of fibrosis that strictly connect the prognostic value of PALS and AF itself.^19^, ^20^

In a different cohort of patients with HFrEF, worse PALS was related to advanced severity of HF disease^19^, ^21^ and even to worse LV systolic and diastolic function. The superior prognostic role of PALS over other clinical and echocardiographic predictors of prognosis, like LAVI, LVGLS and LV filling pressure, was demonstrated.^22^ This was also confirmed in recent machine‐learning approaches.^23^ This parameter has also showed to be associated with HF symptoms improvement after therapy.^24^ AF and ischaemic aetiology may negatively impact both on LA strain and HF prognosis, due to fibrotic changes happening in the LA consequent to these two conditions. Therefore, these should be considered as potential determinants of LA strain in future studies.

In our meta‐analysis, a reduction of PALS of 1% indicated 5% risk of all‐cause mortality and HF hospitalization. This is of very strong impact, because the average normal values of PALS are >39%,^22^ but it is easy to find a relative reduction >5%–10% from normal values, even in patients with first HF events, and in patients with high grades of LA fibrosis we often found values of PALS lower than 10%. This translates, for example, in a higher risk of poor outcome of 50% for patients with a relative 10% reduction of PALS.

This highlights the potential importance of evaluating global PALS both in outpatients and inpatients with HF, which is high feasible thanks to its increasing availability in bigger and smaller centres, the existence of dedicated softwares to LA, and its low time‐consumption. It may represent an additional parameter to estimate prognosis in these patients, aiding clinicians in decision making for more aggressive therapies or stricter follow‐up procedures. As future direction, the re‐evaluation of the prognostic value of global PALS in light of the new therapies for HF may be of interest, since these drugs have shown to induce an early remodelling in HFrEF, HFmrEF and HFpEF, easily detected and predicted by speckle tracking parameters.^24^, ^25^, ^26^

## Limitations

Although this meta‐analysis presents promising results for the introduction of LA strain in daily clinical practice, there are some limitations to acknowledge: the little number of studies included, however with an overall high number of patients and, being highly selected, these studies were homogeneous in study methods and cohort characteristics, limiting some bias. The reported *I* ^2^ value (89%) indicates a high level of heterogeneity, likely due to differences in patient populations, baseline characteristics and clinical settings across studies. While we applied a random‐effects model to account for between‐study variance, this approach does not eliminate within‐study heterogeneity. A sensitivity analysis was not performed due to data limitations. Therefore, the results should be interpreted with caution, as the high heterogeneity may affect the generalizability and validity of the pooled estimates. Future studies with more homogeneous patient populations and standardized methodologies are needed to confirm these findings.

The comparison with basic echocardiographic parameters of LV diastolic function (such as LAVI, E/E', sPAP, PA coupling) was not possible due to the absence of these data in each study. However, studies reporting the HR for these parameters showed lower values than those reported for global PALS (such as LAVI reported HR = 1.02 [1.01–1.03], *P* < 0.001 at univariate analysis [12]; HR = 1.01 [0.99–1.02], *P* = 0.18 at multivariate analysis [2]), and many of these parameters were analysed in the adjusted models of all studies showing the prognostic significance of PALS.

Another limitation is represented by the speckle tracking technique itself, which is not currently available in all centres and for its dependence on acoustic window. However, its availability is increasing, and the introduction of LA‐dedicated software makes the calculation of LA strain more precise and reliable also in suboptimal acoustic windows. Then, the STE software installation on the echocardiographic machine for online measurement, provides a minimal time‐consumption and higher feasibility also in acute settings.

## Conclusions

The prognostic evaluation of patients with HF is currently challenging, particularly in patients with severe impairment of LV function, for whom the management may be more insidious.

The results of our meta‐analysis and meta‐regression analysis showed that global PALS by speckle tracking echocardiography may be used as a reliable prognostic tool in HF, both in acute and chronic setting and especially in older patients with lower LV systolic function, providing an additional independent value for risk stratification.

This added more objective evidence about the relationship between lower PALS and different adverse outcomes, suggesting its use it in clinical practice as additional parameter to guide pharmacological treatment strategies with the aim to improve patients' short‐ and long‐term outcome and quality of life.

## Conflict of interest

None declared.

## Funding

None.

## Data Availability

The data underlying this article will be shared on reasonable request to the corresponding author.
